# Toxicological assessment of multi-walled carbon nanotubes combined with nonylphenol in male mice

**DOI:** 10.1371/journal.pone.0200238

**Published:** 2018-07-20

**Authors:** Hao Fang, Yibin Cui, Zhuang Wang, Se Wang

**Affiliations:** 1 Collaborative Innovation Center of Atmospheric Environment and Equipment Technology (AEET), School of Environmental Science and Engineering, Nanjing University of Information Science and Technology, Nanjing, China; 2 Nanjing Institute of Environmental Sciences, Ministry of Environmental Protection, Nanjing, China; Helmholtz Zentrum Munchen Deutsches Forschungszentrum fur Umwelt und Gesundheit, GERMANY

## Abstract

Carbon nanotubes have attracted increasing attention attributable to their widespread application. To evaluate the joint toxicity of multi-walled carbon nanotubes (MWCNTs) and nonylphenol (NP), we investigated the toxicological effects of NP, pristine MWCNTs, and MWCNTs combined with NP in male mice. After exposing male mice by gavage for 5 days, intracellular superoxide dismutase (SOD) and glutathione peroxidase (GSH-Px) activity, as well as malondialdehyde (MDA) and glutathione (GSH) levels in tissues were determined to evaluate *in vivo* oxidative stress. In addition, genotoxicity was assessed by examining DNA damage in mouse liver and sperm via the comet assay, and transmission electron microscopy (TEM) was used for direct visual observations of mitochondrial damage in the liver. Results from the oxidative damage and DNA damage experiments indicate that after adsorbing NP, MWCNTs at a high dose induce oxidative lesions in the liver and cause DNA damage in mouse sperm; these data offer new insights regarding the toxicological assessment of MWCNTs.

## Introduction

There has been a rapid growth in the application of nanoscale materials, attributable to the development of nanotechnologies [1]. Carbon nanotubes (CNTs), which were discovered in 1991 [2], have attracted a great deal of attention owing to their unique structural, electrical, and mechanical properties, which make them potentially useful in extremely small-scale biological, electrical, and mechanical applications. Evidence suggests that CNTs adsorb organic pollutants effectively. For instance, CNTs adsorb environmental endocrine disruptors (EEDs), neutral dissolved organic matter, and trihalomethanes more extensively than activated carbon materials do [3–5]. Therefore, CNTs are potential adsorbents of these organic pollutants.

Nonylphenol (NP) is a xenobiotic compound used globally to manufacture nonylphenol ethoxylate surfactants and is widely known as a type of EED [6]. Because of its low solubility and high hydrophobicity, NP accumulates under certain environmental conditions, such as in wastewater treatment plants, river water, sediments, and soils. For example, NP can be detected in the surface water of the Daliao river estuary of China, with concentrations ranging from 83.6 to 777 ng/L [7]. It has also been reported that NP exists in pork, chicken, and beef, with concentrations ranging from 0.50 to 0.67 mg/kg [8, 9]. Previous studies have confirmed that NP may interfere with animals and various types of cells more than other EEDs via several mechanisms, including increased proliferation of mammary gland cells [10], production of telemetric associations and chromosomal aberrations [11], irreversibly influencing the reception of fear-provoking stimuli in male rats at a low dosage of 0.1 mg/kg/day [12], inhibiting the growth and differentiation of murine neural stem cells and inducing apoptosis [13], and inducing oxidative stress [14] and reproductive toxicity in Muridae animals [15]. In particular, much attention was paid to NP-induced disruption of male reproductive toxicity, such as reduced testis and epididymis weights as well as decreased sperm count and motility [15, 16].

Although studies have shown that electrochemical-assisted adsorption on multi-walled CNTs (MWCNTs) removes 4-nonylphenol (4-NP) efficiently [17], there is growing concern regarding the safety of CNTs [18–21]. Intense investigations of the adverse health effects have focused on CNT toxicity both *in vivo* and *in vitro*. To date, several *in vivo* studies have shown that CNTs may induce various toxicities, including an increase in the inflammatory response, oxidative stress, granuloma formation, and fibrosis [22–25]. *In vitro* investigations have confirmed these physiological and biochemical responses and provide further support to explain the increased incidence of oxidative stress in cells after exposure to CNTs [26–30]. In addition, NP adsorption on MWCNTs facilitates its bioavailability in the earthworm (*Eisenia fetida*) and increases ecological risks [31]. A recent study indicates that MWCNTs cause toxicity to the invertebrate, *Daphnia magna*, in water [32]. However, to date little is known about the environmental health risks resulting from NP adsorption on MWCNTs.

In this study, CD-1 (ICR) mice were exposed to 4-NP, MWCNTs, or 4-NP adsorbed on MWCNTs (MWCNTs+NP) by gavage; *in vivo* oxidative effects and genotoxic responses to stress, including intracellular superoxide dismutase (SOD) and glutathione peroxidase (GSH-Px) activity, as well as malondialdehyde (MDA) and glutathione (GSH) levels were evaluated. We assessed the genotoxic response by measuring DNA damage in mouse liver and sperm via the comet assay. Further, transmission electron microscopy (TEM) was used for direct visual evidence of mitochondrial damage in liver cells. The objective of this study was to evaluate oxidative and genotoxic effects of NP adsorbed on MWCNTs in mice, along with potential mechanisms.

## Materials and methods

### Materials

MWCNTs made via chemical vaporization deposition (CVD) were obtained from Shenzhen Nanotech Port Co. Ltd. (Shenzhen, China). Morphology and specific surface area of the MWCNTs were determined via TEM (H-7500, Hitachi, Japan) and multipoint Brunauer-Emmett-Teller (BET) analysis, respectively. 4-NP was purchased from Acros Organics Co. Ltd. (New Jersey, USA), and purity was greater than 99%.

### Animals and exposure procedure

CD-1 (ICR) male mice (18 ± 2 g, 4 weeks) were purchased from Beijing Vitalriver Experimental Animal Technology Co. Ltd. (Beijing, China). The animals were housed and maintained on a commercial pellet diet, given distilled water *ad libitum*, and kept in plastic cages in a ventilated animal room. Room temperature was controlled at 22 ± 1°C and relative humidity was maintained at 60 ± 10%, with a 12 h light/dark cycle. The mice were acclimated to this environment for 1 week. All experimental animal procedures were approved by the Ethics Committee of Nanjing University of Information Science and Technology. Animal management was performed strictly in accordance with the standards of the Animal Ethics Committee of Nanjing University of Information Science and Technology. All sections of this report adhere to the ARRIVE Guidelines for reporting animal research [33]. A completed ARRIVE Guidelines Checklist is included in S1 Checklist.

The animals were randomly divided into five groups of five animals each as follows: (1) normal saline control group (CK group), (2) dimethyl sulfoxide (DMSO) vehicle control group (DMSO group), (3) 4-NP treatment group administered 5 mg/kg body weight (NP group), (4) MWCNTs treatment group administered 100 mg/kg body weight (MWCNTs group), and (5) 100 mg/kg MWCNTs+NP treatment group administered 5 mg/kg (MWCNTs+NP group). In addition, 100 μmol/L hydrogen peroxide (H_2_O_2_) was used as a positive control to ensure that the comet assay was functioning properly. For the NP group, 50 mg 4-NP was dissolved in 100 ml DMSO. Since DMSO has been reported to have low toxicity in mice [34], it is necessary to set a DMSO vehicle control group. Materials in the MWCNTs+NP group were pretreated as previously described, with some modifications [31]. Briefly, 20 mg 4-NP was dissolved in 100 ml DMSO; 200 mg MWCNTs was then added to the solution. The mixture was placed into a water-bathing constant temperature vibrator (THZ-82, Changzhou, China) and vibrated at 150 rpm for 24 h. After standing for 24 h, the mixture was centrifuged at 21000 × *g* for 10 min and the concentration of 4-NP was measured in the supernatant using high performance liquid chromatography (HPLC, Alliance 2695, Waters, USA) [35]. Results indicate that 50 mg/g 4-NP adsorbed on the MWCNTs. The MWCNTs+NP were collected (100 mg), dried, and administered to the MWCNTs+NP group. Prior to animal treatment, the MWCNTs and MWCNTs+NP were suspended in normal saline and dispersed by ultrasonic vibration for 15 min. Suspensions were subjected to dynamic light scattering (DLS) spectroscopy (ZEN 3600, Malvern, UK) for the characterization of size and dispersity. Particle size distributions were determined on the basis of number, volume, and scattering intensity [36]. All animals were treated by gavage of a 0.2 ml suspension once per day for 5 days.

After 5 days, no adverse event was found in any of the experimental groups. The mice were sacrificed and the liver, kidneys, heart, spleen, and lungs from each group were collected to evaluate intracellular oxidative damage. The liver and sperm were also collected to assess the genotoxic response.

### Analytical procedure

SOD and GSH-Px activity, as well as MDA and GSH levels were measured in each group to determine oxidative damage to the liver, kidneys, heart, spleen, and lungs. Before experimental analysis, each tissue (5 per group) was cut into pieces and mixed with ice-cold 0.86% NaCl to form 10% tissue homogenate. The mixture was then homogenized with an ultrasonic processor (JY-250, Zhejiang, China) and centrifuged at 600 × *g* (4°C) for 15 min. The supernatants were used in the enzymatic assays. The activities of SOD, GSH, GSH-Px and the levels of MDA were determined using commercial assay kits purchased from Nanjing Jiancheng Bioengineering Institute (Catalog number: A001-1, A006-1, A005, A003-1, China). SOD activity was determined using a xanthine-xanthine oxidase and nitro blue tetrazolium (NBT) system. The endpoint of SOD activity was detected based on the presence of red substances in the reaction system by absorbance at 550 nm after 40 min of reaction time at 37°C. One unit of SOD was defined as the amount of protein that inhibited NBT reduction by 50% [37]. MDA levels were assessed using the thiobarbituric acid (TBA) assay [38]. The absorbance of red TBA-MDA complex was determined at 532 nm. GSH reacts with 5, 5’ -dithiobis (2-nitrobenzoic acid) (DTNB) to produce stable yellow substances and the absorbance was detected at 420 nm. The GSH-Px activities were also measured using the assay kit based on the principle that oxidation of GSH and H_2_O_2_ could be catalyzed by GSH-Px to produce oxidized glutathione (GSSG) and H_2_O. The decrease in GSH at 412 nm during the 5 min of reaction time at 37°C indicates GSH-Px activity in tissues. The MDA and GSH content as well as the GSH-Px activity were calculated per the detailed instructions on the assay kits. All the enzymes and MDA contents were detected using a spectrophotometer (UV1102Ⅱ, Techcomp, Shanghai, China).

The comet assay was performed according to the method described by Singh et al., with some modifications [39]. After the mice were sacrificed, the livers and testicles were obtained in 35-mm glass plates and washed twice with phosphate-buffered saline (PBS). These two tissues were transformed into a 10-ml beaker and cut into small pieces; then, the liver and sperm cells were collected through a 150-μm mesh. Cell suspensions were centrifuged at 860 × *g* for 3 min and cells were re-suspended in PBS. Prior to the comet assay, a trypan blue dye-exclusion staining assay was used to ensure that cell viability was greater than 95%. Electrophoresis was conducted at 4°C for 20 min at 25 V and 100 mA in the dark. Slides were then stained with ethidium bromide (EB) and scored using a fluorescent microscope (BX41, Olympus, Japan). Fifty images were randomly selected for each group and analyzed with CASP software per the method described by Collins et al. [40].

Liver samples were fixed in 2.5% glutaraldehyde and embedded as sections following routine techniques for TEM observations (H-7650, Hitachi, Japan) [41].

### Statistics

The data are presented as means ± SD (standard deviation). Statistically significant differences among treatment groups were determined using one-way analysis of variance (ANOVA), followed by Tukey’s honest significant difference (HSD) *post hoc* test (equal variances) or Dunnett’s T3 *post hoc* test (unequal variances). All the above tests were performed using SPSS 16.0 software. Differences were considered statistically significant when the *p*-value was less than 0.05.

## Results

### Characteristics of MWCNTs

**Fig 1:**
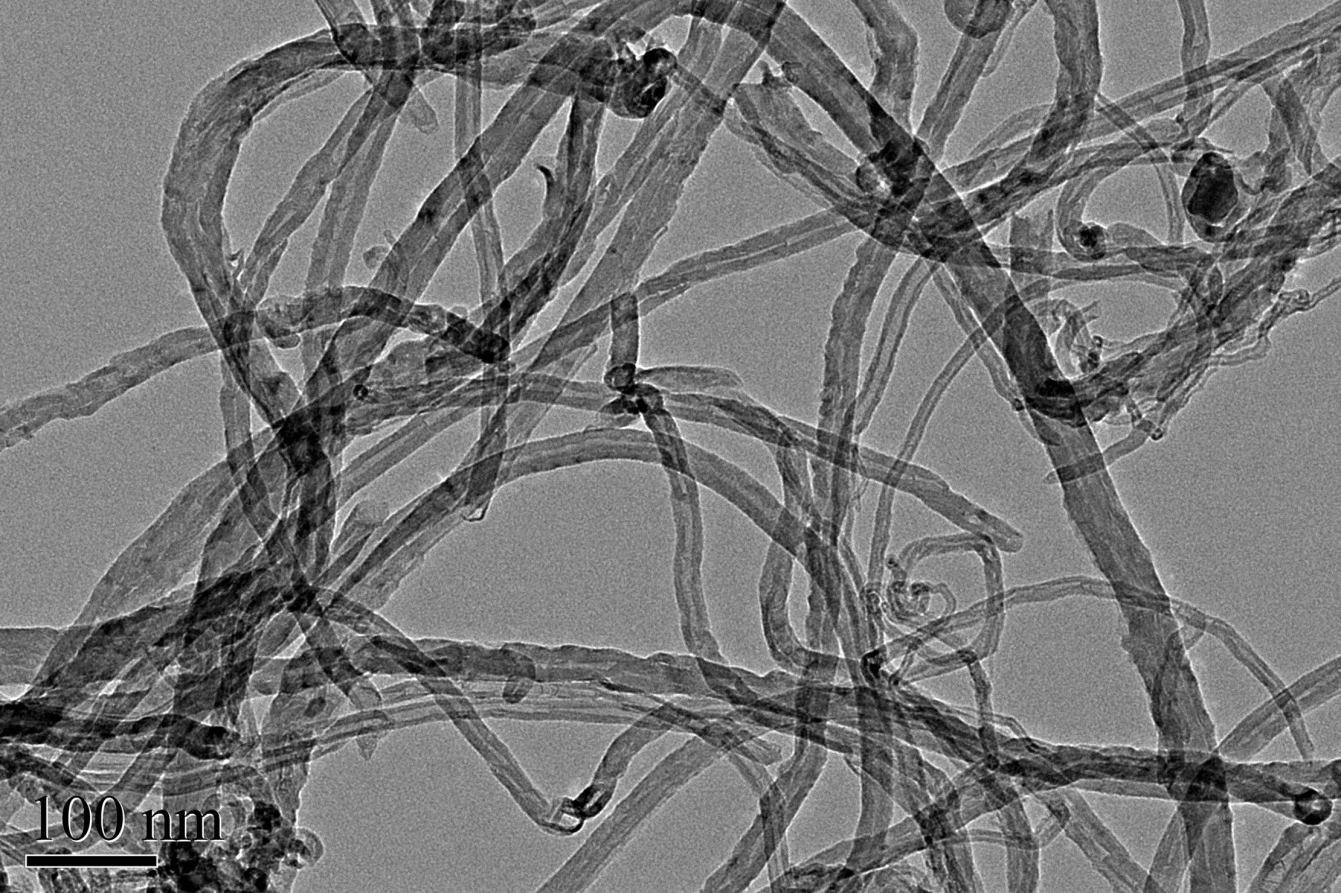
Transmission electron microscopy (TEM) micrograph of multi-walled carbon nanotubes (MWCNTs).

The MWCNTs were 10 μm (Fig 1) in length, with a 10–20 nm outer diameter and purity greater than 99%. The special surface area of the MWCNTs was 500 m^2^/g according to BET analysis.

### Characterization of MWCNTs and MWCNTs+NP in dispersion

DLS measurements of MWCNTs and MWCNTs+NP suspensions showed that their size distribution ranged from 37 nm to 550 nm, with an average hydrodynamic size of 125 nm and 137 nm, respectively (S1 Fig).

### Oxidative damage in the liver, kidneys, lungs, heart, and spleen of male mice

**Fig 2:**
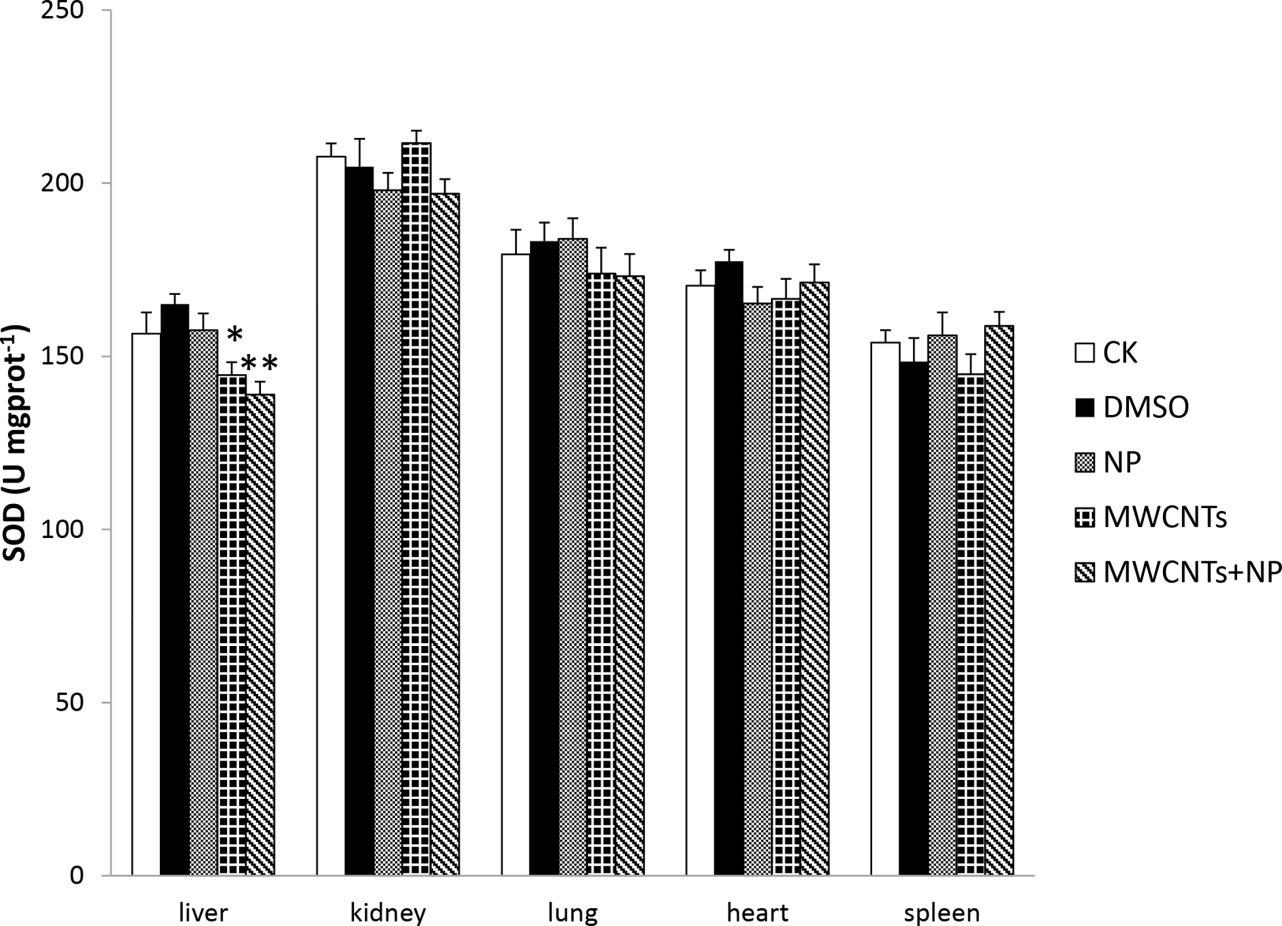
Superoxide dismutase (SOD) activity in the organs of mice. Results are expressed as the means ± SD (n = 5). Significant differences from the control (CK) group are denoted by * *p* < 0.05 and ** *p* < 0.01.

**Fig 3:**
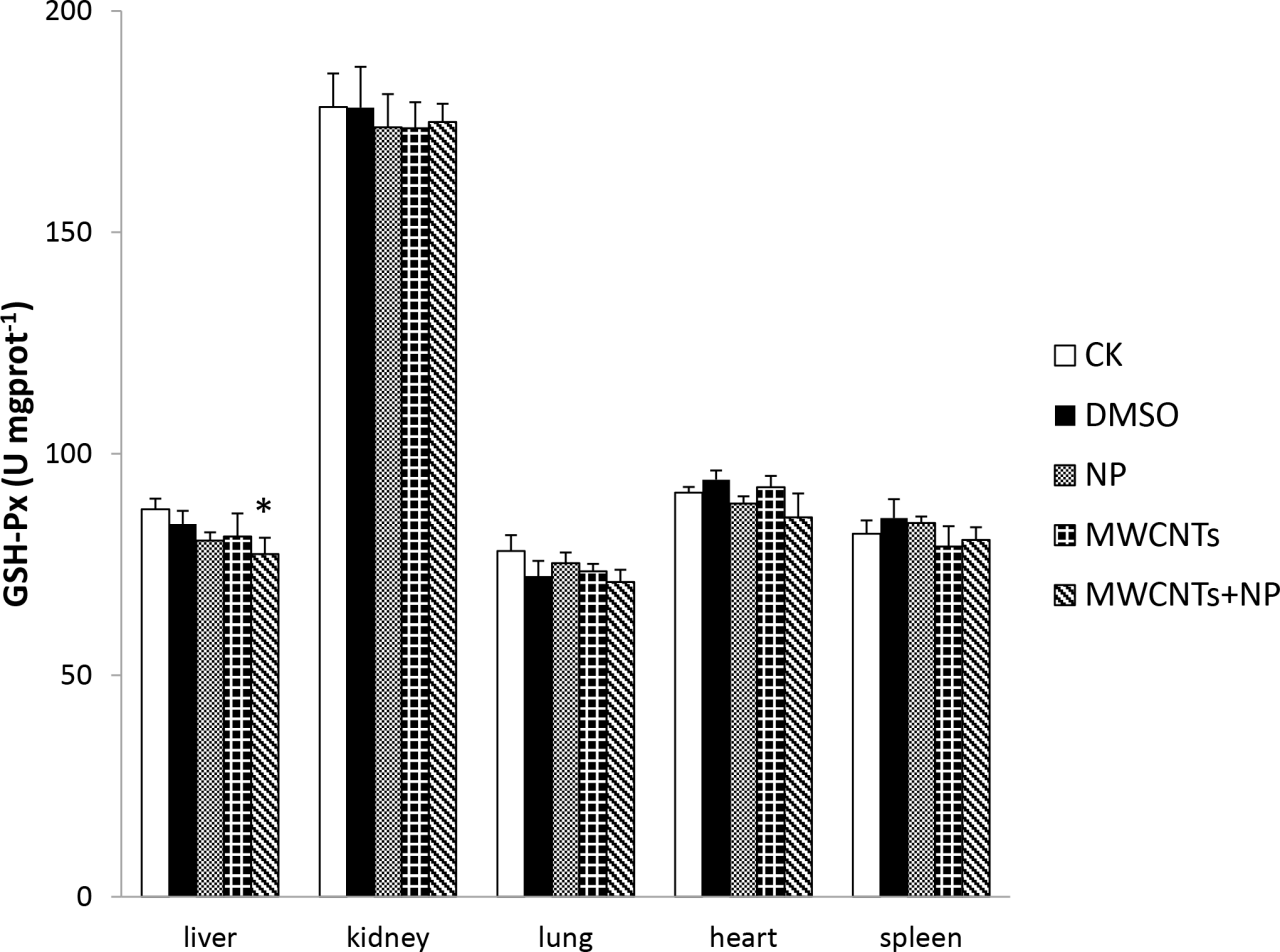
Glutathione peroxidase (GSH-Px) activity in the organs of mice. Results are expressed as the means ± SD (n = 5). Significant differences from the control (CK) group are denoted by * *p* < 0.05.

**Fig 4:**
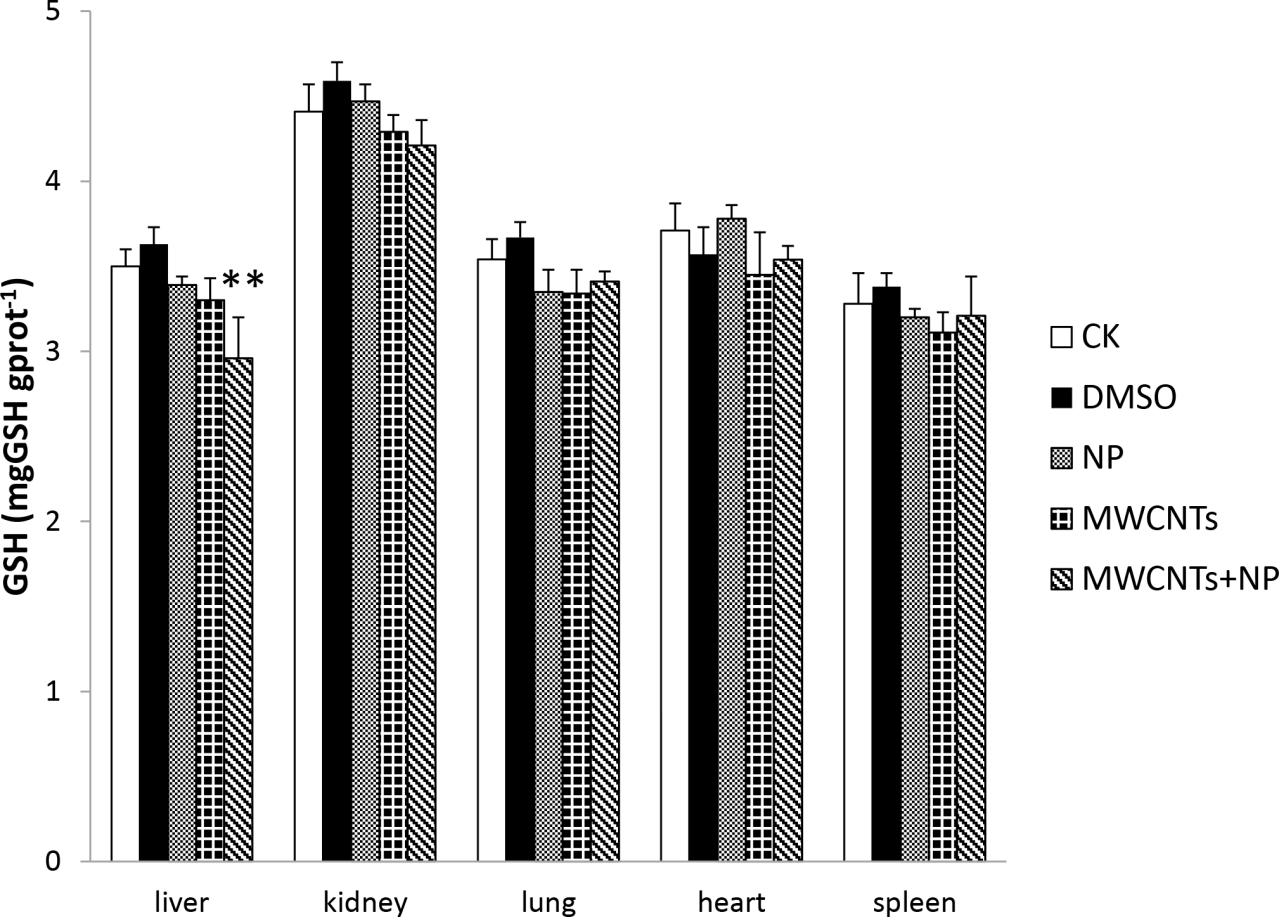
Glutathione (GSH) levels in the organs of mice. Results are expressed as the means ± SD (n = 5). Significant differences from the control (CK) group are denoted by ** *p* < 0.01.

**Fig 5:**
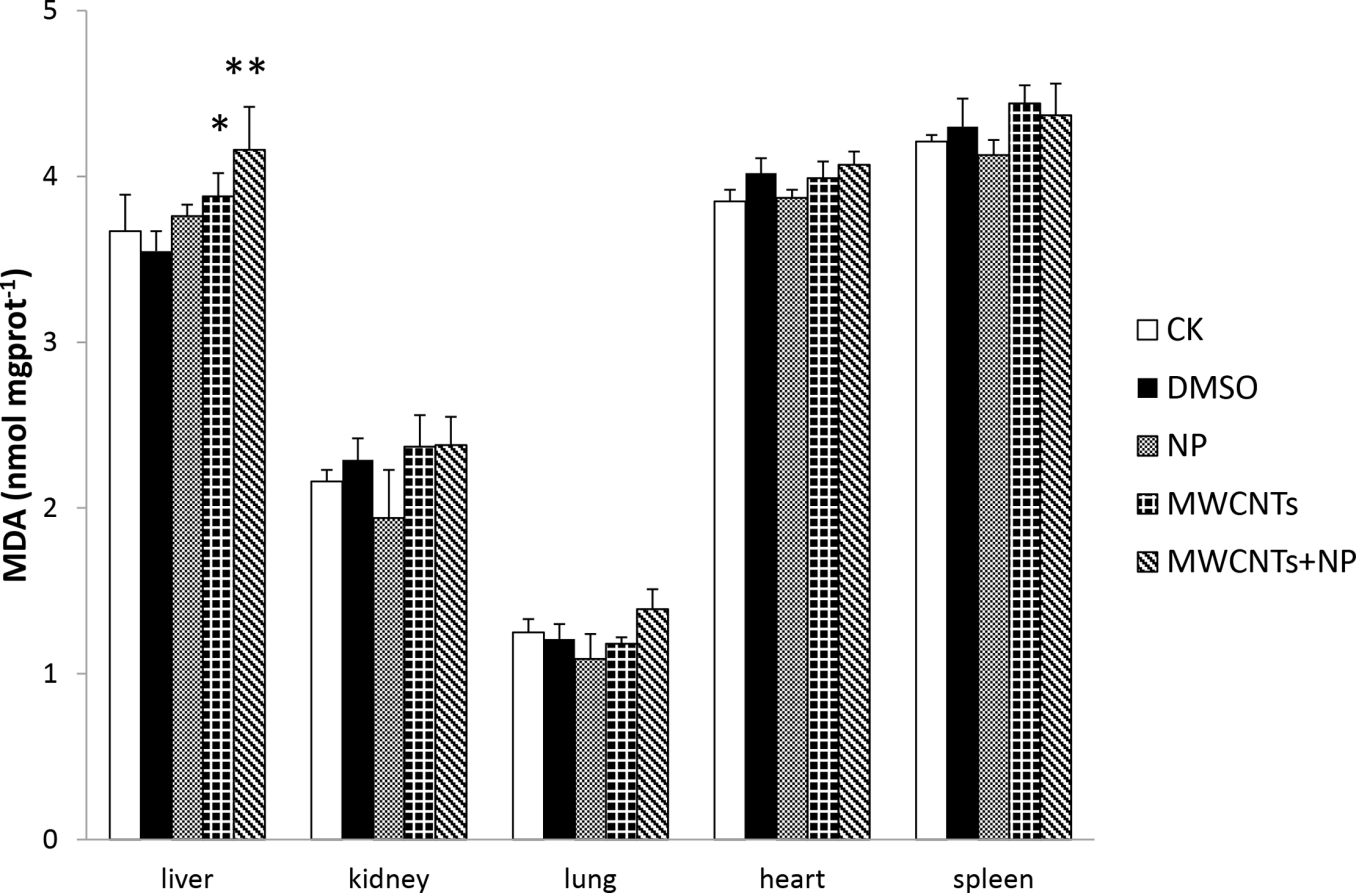
Malondialdehyde (MDA) content in the organs of mice. Results are expressed as the means ± SD (n = 5). Significant differences from the control (CK) group are denoted as * *p* < 0.05 and ** *p* < 0.01.

To investigate the intracellular response to MWCNTs+NP, several anti-oxidative enzymes and antioxidants were evaluated. SOD and GSH-Px activity, as well as GSH and MDA content in the liver, kidneys, lungs, heart, and spleen of mice are shown in Figs 2–5. No significant differences were observed in antioxidative enzyme activity or antioxidant levels in the five organs among the treated and control groups, except for that in the liver. SOD activity in the liver significantly decreased (*p* < 0.05) after administration of MWCNTs; however, this effect was attenuated in the MWCNTs+NP group (*p* < 0.01) (Fig 2).

GSH-Px enzymes can protect organs from oxidative damage by consuming H_2_O_2_ *in vivo* and can be used as an indicator of intracellular oxidative stress. Liver GSH-Px activity in the MWCNTs+NP group was lower (*p* < 0.05) than that in the CK group (Fig 3). In addition, GSH depletion in the liver was significantly different (*p* < 0.01) from the CK group after exposure to MWCNTs+NP (Fig 4).

We measured MDA content in the organs to determine the extent of lipid peroxidation. MDA content was higher in the livers from the MWCNTs group (*p* < 0.05) than that in the livers from the CK group and relatively higher (*p* < 0.01) than that in the livers from the MWCNTs+NP group (Fig 5).

### DNA damage in mouse liver and sperm

**Fig 6:**
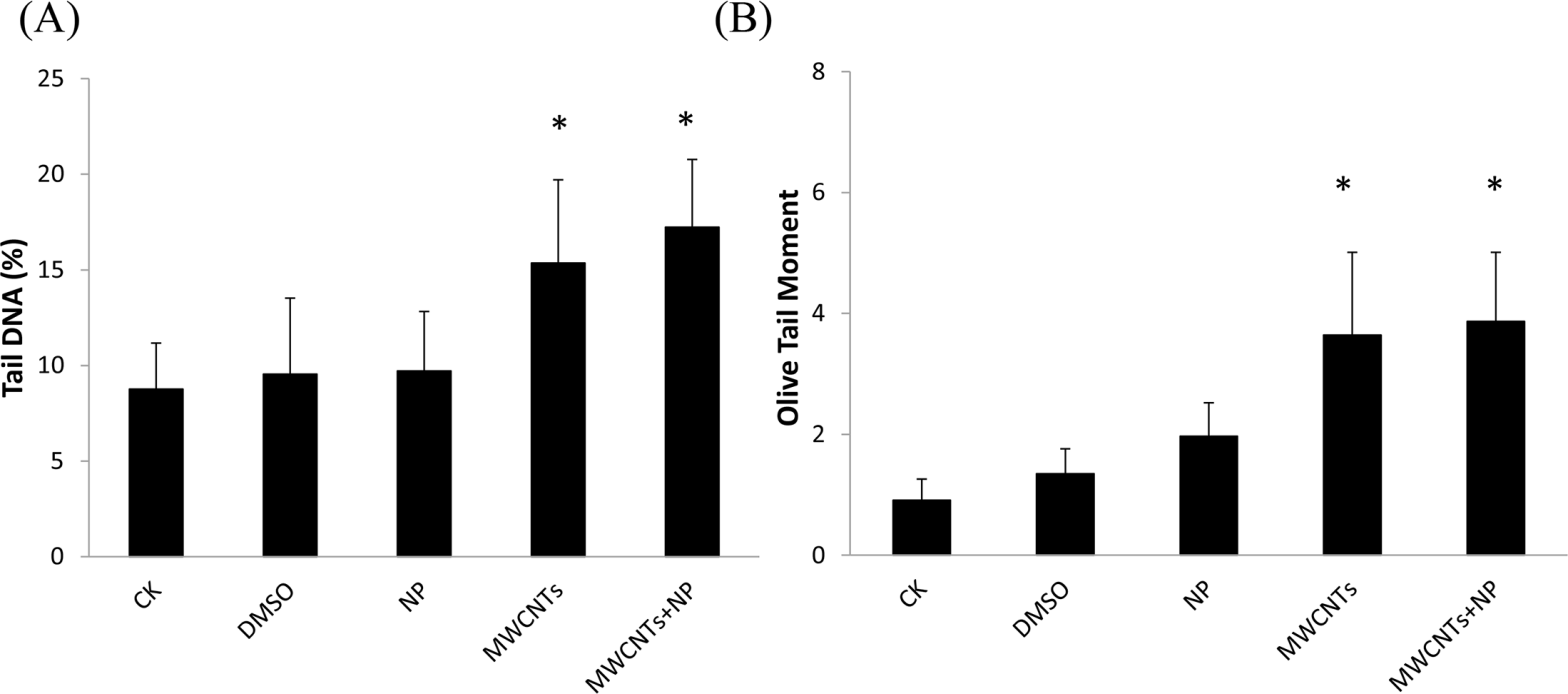
Tail DNA (A) and olive tail moment (OTM) (B) from the comet assay in mouse liver after exposure to saline only (CK control), dimethyl sulfoxide (DMSO), 4-nonylphenol (NP), multi-walled carbon nanotubes (MWCNTs), and MWCNTs+NP. The values are means ± SD (n = 5). Significant differences from the CK group are denoted by * *p* < 0.05.

**Fig 7:**
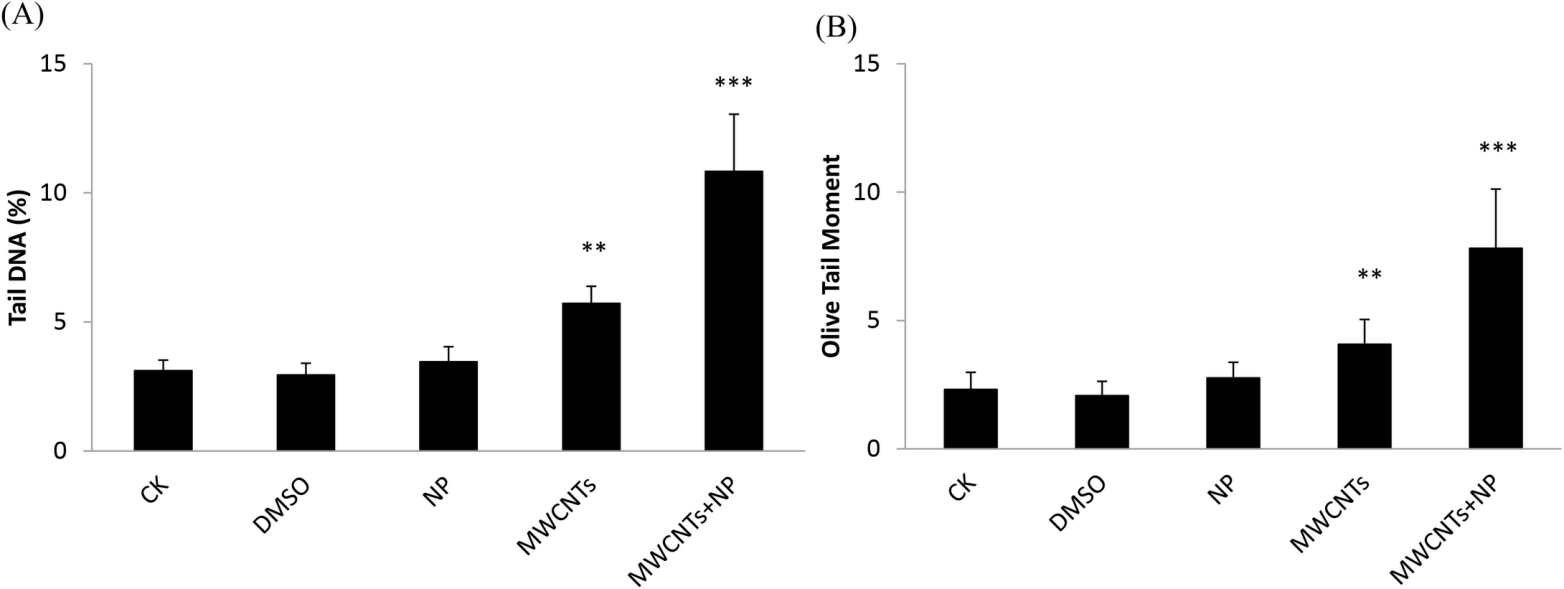
Tail DNA (A) and olive tail moment (OTM) (B) from the comet assay in mouse sperm after exposure to saline only (CK control), dimethyl sulfoxide (DMSO), 4-nonylphenol (NP), multi-walled carbon nanotubes (MWCNTs), and MWCNTs+NP. The values are means ± SD (n = 5). Significant differences from the CK group are denoted by ** *p* < 0.01 and *** *p* < 0.001.

To evaluate DNA damage in the liver and sperm, tail DNA and olive tail moment (OTM) were determined via the comet assay; the results are shown in Figs 6 and 7. DNA damage in mouse liver after exposure to MWCNTs and MWCNTs+NP was significantly different from that in mouse liver from the CK group (Fig 6, *p* < 0.05), while DNA damage in mouse sperm was higher after exposure to MWCNTs and MWCNTs+NP (Fig 7, *p* < 0.01, *p* < 0.001,respectively). No significant difference in DNA damage in the liver and sperm was observed in the DMSO and NP groups.

### Liver mitochondrial damage in mice

**Fig 8:**
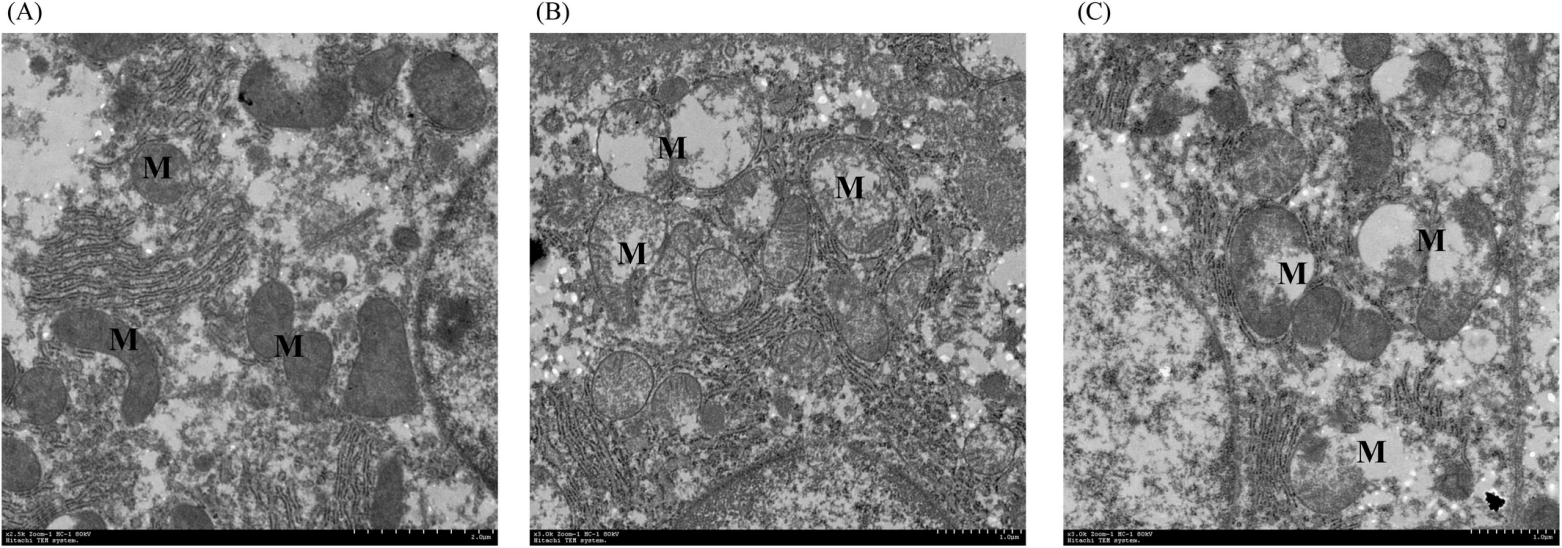
Transmission electron microscopy (TEM) images of mouse liver cells after exposure to saline (CK control) (A), multi-walled carbon nanotubes (MWCNTs) (B), and MWCNTs+NP (C). M represents the mitochondria.

We used TEM to investigate the comparative effects of MWCNTs and MWCNTs+NP treatment on cellular structures and organelles in mouse livers. Five days after exposure, 100 mitochondria were randomly selected to check the morphology in each animal and the results showed that mitochondrial abnormalities, including reduction, disorganization, and fractures (Fig 8B and 8C), were significantly greater in the MWCNTs and MWCNTs+NP groups than in the CK group (Fig 8A).

## Discussion

In this study, we compared the anti-oxidative damages of NP, pristine MWCNTs, and MWCNTs+NP in mice. In addition, we evaluated genotoxic effects using the comet assay during acute toxicity tests. Although no differences in body weight and organ coefficients were observed in all five groups (data not shown), the enzyme and genotoxic results indicate that MWCNTs+NP exposure for 5 days in male mice results in greater toxicity than exposure to NP or MWCNTs alone. As a common mechanism for intracellular damage, oxidative stress has been clearly implicated in the induction of inflammation in many studies examining CNTs both *in vivo* and *in vitro* [1, 27, 42]. CNTs stimulate the generation of reactive oxygen species (ROS), which can damage lipids, carbohydrates, proteins, and DNA [43, 44]. ROS-mediated toxicity has also been observed *in vitro* for single-walled CNTs with a diameter of 8 nm and length of < 5 μm [30]. Normally, antioxidant enzymes such as SOD and GSH-Px reduce H_2_O_2_ and superoxide radicals, protecting polyunsaturated fatty acids from lipid peroxidation and further preserving the structure of the cell membrane. However, excess ROS production destroys the natural antioxidant defense system and leads to several sub-cellular injuries, including protein denaturation, membrane damage, and DNA damage [1]. In the present study, we investigated changes in SOD and GSH-Px activity, as well as GSH levels to compare the toxicity of NP, MWCNTs, and MWCNTs+NP. Our results suggest that MWCNTs+NP induce significant changes in GSH-Px activity and deplete GSH in the liver, although evidence suggests that pristine CNTs are not toxic or little toxic to animals when administered by gavage (50 mg/kg) or by the intraperitoneal route (250 mg/kg) [45, 46]. In this study, decreased SOD levels in the liver were observed in both the MWCNTs and MWCNTs+NP groups. This is likely attributable to the high concentration of MWCNTs (100 mg/kg) used in this study. It has been reported that the oral no-observed-adverse-effect-level value of NP seems to range from approximately 50 to 100 mg/kg [47]. In this study, we set a high concentration of MWCNTs (100 mg/kg) and a relatively low dose of NP (5 mg/kg). This is mainly due to the limitation of adsorbing capacity of NP on MWCNTs in our pilot study, which indicated that the largest extent of NP adsorption on MWCNTs was approximately 58 mg/g. In the meanwhile, although previous study showed animals were successfully administered via the intraperitoneal route by using an extremely high dose (250 mg/kg) [46], in this study we still chose the oral route for the sake of safety. The oxidative damage appeared to be higher in the 100 mg/kg MWCNTs+ 5 mg/kg NP group than in the other treatment groups. The adsorptive properties of MWCNTs may explain this phenomenon. The addition of NP to the MWCNTs may have exacerbated the induction of intracellular ROS generation by simultaneously exerting adverse effects on the antioxidant defense system. Although studies have suggested that NP is an environmental contaminant that results in adverse environmental health effects attributable to oxidative stress both *in vivo* and *in vitro* [14, 48–50], in our study, there were no significant differences in toxicity between the NP and CK groups. We inferred from these data that the NP exposure dose and time used in this study were not sufficient to stimulate ROS generation and induce oxidative damage; however, when NP adsorbs to MWCNTs, it remains in the tissues for a longer duration.

MDA is a major peroxidation product that is formed under conditions of oxidative stress and can be used as an indicator of lipid peroxidation [51]. Instability of the plasma membrane results from active oxygen atoms generated by peroxide. It has been reported that most MWCNTs are excreted in the feces when administered to mice by gavage [52], and an *in vitro* study suggested that MWCNTs are not taken up by enterocytes [53]; however, in this experiment, significant increases in liver MDA levels in the MWCNTs and MWCNTs+NP groups were observed. In addition, MDA content in the MWCNTs+NP group was higher than that in the MWCNTs group. In combination with the observed changes in antioxidant enzyme activity, the MDA results suggest that most of the oxidative stress occurred in the liver of MWCNTs+NP-treated mice. We inferred from these data that some MWCNTs enter the circulatory system via the gastrointestinal tract, resulting in liver damage.

The comet assay is a useful tool for studying the genotoxic effects of CNTs [29, 54]. Currently, the genotoxic potential of CNTs is not clear, attributable to differences in experimental design among studies, including the various models used, exposure routes, type of CNTs examined, administered concentrations, and assessed endpoints. Genotoxic responses may transpire via direct mechanical injury or as a secondary result of CNT-mediated ROS generation and oxidative stress [55]. The results of the comet assay in the present study clearly show DNA damage were observed in the liver and sperm from mice administered MWCNTs. It was reported that repeated intravenous injections of water-soluble MWCNTs to male mice (5 mg/kg) can cause reversible testis damage without affecting fertility [56]. Further, DNA damage were highest in the liver and sperm of mice administered MWCNTs+NP. Although the investigation revealed that adsorption of another endocrine disruptor, bisphenol A (BPA), to a CNT (with the highest dose of 2.4 mg/kg BPA and 65 mg/kg carboxylated MWCNTs) reduced its endocrine disrupting effect in mice male offspring [57], we consider that the properties of MWCNTs+NP may differ from that of MWCNTs+BPA after adsorption. Another possible explanation is that MWCNTs+NP accumulate in mouse liver and sperm mitochondria [58]. Previous studies have already demonstrated that NP could lead to reproductive toxicity in Muridae animals such as rats [59] and mice [15, 60], and cause abnormal conditions including decreased testis weights and sperm motility, reduced SOD and GSH levels as well as increased MDA contents in the reproductive organs. As there was no significant difference in the DNA damage between the NP group and the CK group, we inferred that it is due to a short exposure time and low dose (5 mg/kg) used in this study. On the contrary, MWCNTs act as a carrier for NP, which then persists in the liver and sperm, causing additional DNA damage.

It was reported that NP placed in direct contact with the liver could lead to more gene activation than that caused by estradiol, indicating that tissue-specific effects should also be considered [61]. Since the liver suffered more oxidative damage from MWCNTs+NP and MWCNTs than other organs, TEM was used to directly observe mitochondrial damage in the liver. Disorganization and fractures in mitochondria with broken cristae and membrane were observed in the liver tissues treated with MWCNTs (Fig 8B), while these lesions were more severe in the liver tissues treated with MWCNTs+NP (Fig 8C), indicating that both exposure induce hepatic mitochondrial damage. This result provides direct evidence that mitochondria are candidate organelles for studying the toxicity of MWCNTs and MWCNTs+NP administered at high doses.

## Conclusions

To investigate the toxicological effects induced by NP, MWCNTs, and MWCNTs+NP in mice, several anti-oxidative defense system parameters were examined, with the comet assay used specifically to study genotoxicity. No obvious acute toxicity was observed 5 days after exposure to NP at a dose of 5 mg/kg in mice. In addition, high doses of MWCNTs+NP induced more oxidative lesions in the liver and caused more DNA damage in the sperm than pristine MWCNTs, as shown by measuring changes in markers of oxidative damage and via the comet assay.

## Supporting information

S1 Checklist Completed “The ARRIVE Guidelines Checklist” for reporting animal research in this manuscript. (PDF)

S1 Fig Dynamic light scattering (DLS) spectra of MWCNTs and MWCNTs+NP suspensions. (TIF)
